# Model Predictive Control via Output Feedback Neural Network for Improved Multi-Window Greenhouse Ventilation Control

**DOI:** 10.3390/s20061756

**Published:** 2020-03-22

**Authors:** Dae-Hyun Jung, Hak-Jin Kim, Joon Yong Kim, Taek Sung Lee, Soo Hyun Park

**Affiliations:** 1 Smart Farm Research Center, Korea Institute of Science and Technology (KIST), Gangneung-si, Gangwon-do 25451, Korea; jeoguss@gmail.com (D.-H.J.); tslee@kist.re.kr (T.S.L.); 2 Department of Biosystems and Biomaterial Engineering, College of Agriculture and Life Sciences, Seoul National University, Seoul 08826, Korea; kimhj69@snu.ac.kr (H.-J.K.); tombraid@snu.ac.kr (J.Y.K.)

**Keywords:** greenhouse climate modeling, machine learning, multi-window ventilation, greenhouse climate control

## Abstract

Maintaining environmental conditions for proper plant growth in greenhouses requires managing a variety of factors; ventilation is particularly important because inside temperatures can rise rapidly in warm climates. The structure of the window installed in a greenhouse is very diverse, and it is difficult to identify the characteristics that affect the temperature inside the greenhouse when multiple windows are driven, respectively. In this study, a new ventilation control logic using an output feedback neural-network (OFNN) prediction and optimization method was developed, and this approach was tested in multi-window greenhouses used for strawberry production. The developed prediction model used 15 inputs and achieved a highly accurate performance (R^2^ of 0.94). In addition, the method using an algorithm based on an OFNN was proposed for optimizing considered six window-opening behavior. Three case studies confirmed the optimization performance of OFNN in the nonlinear model and verified the performance through simulations. Finally, a control system based on this logic was used in a field experiment for six days by comparing two greenhouses driven by conventional control logic and the developed control logic; a comparison of the results showed RMSEs of 3.01 °C and 2.45 °C, respectively. It confirmed the improved control performance in comparison to a conventional ventilation control system.

## 1. Introduction

Greenhouses are a widely used agricultural system that can provide optimal growing conditions for crops, regardless of season, as the controlled inside environment is less affected by exterior weather conditions. Greenhouse crop growth is particularly influenced by CO_2_ level, photosynthetically active radiation, and temperature; the latter two directly affect photosynthesis under diurnal conditions. Maintaining an appropriate temperature is a major concern for greenhouse environmental control because this affects plant development, quality, and production quantity [1,2]; controlling temperature also affects humidity [3,4]. The most important method for maintaining greenhouse temperature is natural ventilation, which mixes external and internal air conditions but is very difficult to artificially control. 

Greenhouses are highly nonlinear and strongly coupled systems that are strongly influenced by weather and the behavior of actuators used for climate control [5]. In recent years, many studies have proposed advanced control methods for greenhouse environments [6,7,8]. Modern greenhouses use multiple-paned windows for more active natural ventilation, but it is more difficult to automatically control these complex systems and determine their effect on ventilation. Models of the window-opening process must consider both external temperature and the effects of wind direction and speed. In addition, it is difficult to continually develop new models for ever-changing configurations of greenhouses and their windows. Therefore, it is necessary to develop an improved ventilation control modeling method that can solve these limitations.

Many studies have designed ventilation control systems through models based on physical phenomena occurring within greenhouses [9,10,11,12]. A similar approach attempts to guide ventilation-based changes in greenhouse environments based on weather or other relevant environmental factors [13,14,15]. These methods use control logic based on modeling material property flow according to the law of conservation of physical energy or experience-based empirical modeling of greenhouses.

The most realistic control algorithms are based on proportional–integral–derivative logic, but it is difficult to apply this effectively in complex systems such as greenhouses because the relevant coefficients must be tuned [16]. Furthermore, it takes the long response time for the actuator to affect the internal environmental variables such as inside temperature and humidity due to greenhouse characteristics, and the influence of other environmental factors is very large. Therefore, most such studies have been conducted through simulations [5,17], and highly sophisticated model development is needed to tune the relevant coefficients to a variety of physical phenomena.

Models based on neural networks are suitable for both linear and nonlinear modeling and have been applied to greenhouse environment modeling and control logic [18,19,20]. Many studies have reported reliable results in environmental prediction modeling using artificial neural networks [18,21,22,23,24]. However, relatively few have used this method to control greenhouse environments and most have been focused on simulations. For example, Fourati and Chtourou [25] adopted an Elman neural network to emulate the dynamics of simulated greenhouse performance using a neural-network-based controller. Fitz-Rodríguez et al. [6] designed a dynamic greenhouse environment simulator for use in utilizing greenhouse control principles. 

In addition, the modeling of greenhouse environmental changes has led to studies investigating the use of predictive control [5,8,26,27]. For example, Blasco et al. [26] assessed model-based predictive control logic and an optimization technique based on a genetic algorithm, with results indicating a wide flexibility in selecting the control objectives. In addition, Coelho et al. [8] applied the particle swarm optimization algorithm to such control logic in a greenhouse air temperature controller and computed outputs to optimize the greenhouse’s future environment. Techniques of input–output data processing based on artificial neural networks are called black-box modeling [17,28]. However, these models require understanding and interpretation of the model to determine the parameters of the model-based controller, and the black-box model needs to be analyzed by an empirical method. In order to determine the parameters of these model-based controllers, it is necessary to understand and interpret the model [29]. For the optimal nonlinear tracking problem, the output feedback neural-network (OFNN)-based approximate techniques have been developed to generate an approximate solution [30]. With this inherent approximation capability of neural networks systems and adaptive neural controllers were proposed for nonlinear systems [31,32,33]. The possibility of applying neural network-based control algorithms in greenhouse environments where mathematical modeling is difficult has been reported [26], but the practical applications are still insufficient. 

This study focused on developing a new approach to applying control logic based on OFNN algorithms for improved control of natural greenhouse ventilation. This is a promising idea based on a model that predicts temperature changes in response to window opening activity in greenhouses. In order to validate the proposed method, the method was applied to a real greenhouse and verified by comparing the results. The next section describes an experimental greenhouse, the design of the proposed method. The results are shown in Section 3, and Section 4 presents the discussion. Conclusions are summarized in Section 5.

## 2. Materials and Methods

### 2.1. Experimental Greenhouse

**Figure 1:**
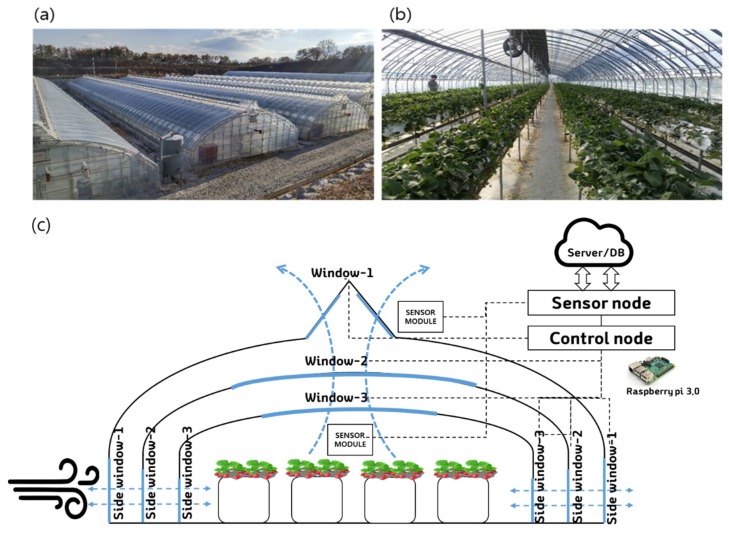
Strawberry greenhouse used in this study: (**a**) exterior and (**b**) inside with multi-window shell structure, and (**c**) schematic of experimental greenhouse monitoring and control system.

The greenhouses used in this study were east–west oriented and single-span greenhouses covered with polyethylene (PE) films. The upper part of the greenhouse was arched and consisted of about 3 layers of wall, which consisted of a width of 7 m, a height of about 3 m, a length of 70 m, and a growing area of about 500 m^2^. This strawberry farm consisted of three greenhouses with the same area as described above, each of which could be controlled independently. Interior and exterior photographs of the greenhouse are shown in Figure 1a,b. The greenhouse was equipped with an automated system, with sensors for monitoring the internal temperature, humidity, and carbon dioxide, and automatically controlled windows and internal fans for temperature control. In the present study, the ventilation control system installed in advance was designed to calculate the ventilation load using a linear algorithm and operated with a P (proportional)-band based algorithm [34]. The accumulated data were obtained by operating a P-band control logic for two months, which had all of the environmental information and control history from March 2018 to May 2018.

The configuration diagram of the greenhouse is shown in Figure 1c. Temperature, relative humidity, and CO_2_ concentration of the inside climate were measured by two sensor modules (SH-VT250, Soha tech, Korea), and the two sensor values were averaged. The sensors were installed at the center of the greenhouse, and the specifications of the sensors are shown in Table 1. The environment controller consisted of a sensor node and a control node for processing sensor data (Figure 1c). The software program operating the individual nodes was installed on a Raspberry Pi (Model B, Raspberry Pi foundation, United Kingdom). Monitoring and control logic were implemented using an open platform program (FarmosV2, Jinong Inc., Gyeonggi-province, Republic of Korea) [35]. The control logic used a control algorithm based on a P-band. The P-band used for ventilation is a system that determines the window opening (%) by calculating the ventilation load through the external temperature, wind direction, wind speed, and solar radiation as the setting values through the linear coefficients. All the control signals and sensor values of the environmental controller were stored in the DB (database) as described above and used for prediction model development and control algorithm design.

The ventilation window structure of the greenhouse was driven by a total of six windows, which were three top windows and three side windows. The top window 1 was located on the outermost side of the greenhouse and had a triangular structure that allowed the wind to flow from both sides. Similarly, in the case of side window 1, it was located on the outermost side and the left and right sides opened simultaneously. Operating ranges of the three side windows were 800 mm, 760 mm, and 650 mm, and they opened proportionally when the ventilation control logic determined that ventilation was necessary. All ventilation windows operated between 0% and 100% of opening and closing proportional to time. Windows 1 and 3 had to be opened for direct mixing with the air inside the greenhouse because of the characteristic of the experimental greenhouse; it was a 3-ply multi-window structure. In the case of window 2, it could be set to determine a more minute ventilation amount, and an additional insulation role was possible.

### 2.2. Neural Network Based Temperature Prediction Model

**Figure 2:**
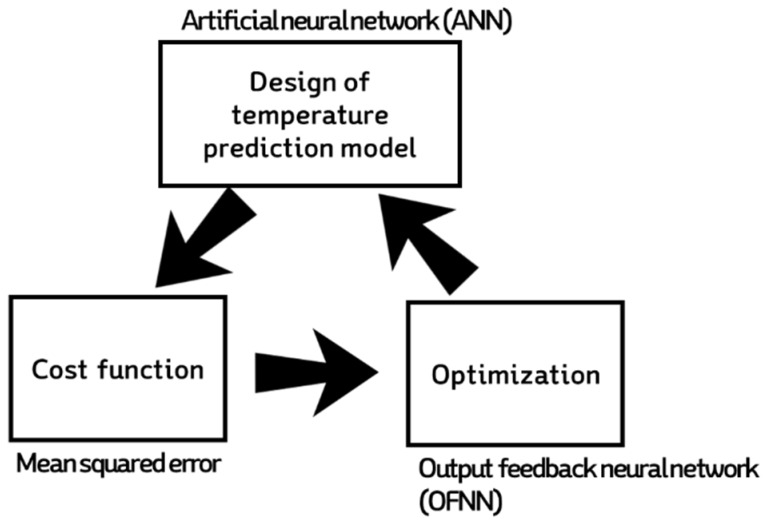
Schematic of the neural-network-based temperature prediction model and optimizer.

**Figure 3:**
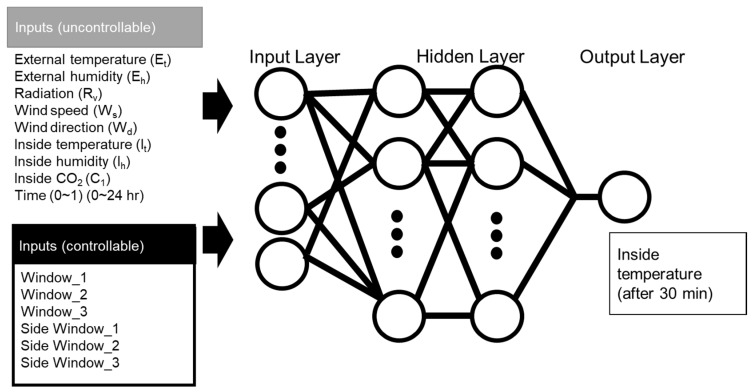
Structure of the neural network model for prediction of the inside temperature.

Our proposed temperature control method was theoretically founded on model-based predictive control logic (Figure 2). First, a prediction model was developed based on data accumulated in the DB, mainly sensor data and actuator history. The prediction model was a black-box model based on an artificial neural network; the output value was the predicted inside temperature after 30 min. The control decision determined the control signal value corresponding to the input of the prediction model feedback from the output of the prediction model to the optimization node. The ANN-based prediction model consisted of four layers of neurons or nodes: the input layer, two hidden layers, and the output layer (Figure 3). Signals entering the input layer were transmitted to the hidden layers and output layer through functions. The model used 15 input variables divided between the data obtained from the sensors and the current controller history. The first hidden layer used 45 nodes and the hyperbolic tangent (Tanh) activation, while the second hidden layer used 30 nodes and the rectified linear unit (ReLU) activation function (Table 2). In the previous study [20], the results were verified through various combinations of active functions in the hidden nodes and parameter values, but no significant correlation was found. For training, the Levenberg–Marquardt algorithm was used, a gradient descent method for avoiding local minima and overfitting [20] of the 73,440 samples used for model development; training used 70%, validation 15%, and testing 15%.

### 2.3. Output Feedback Neural Network

Our ventilation control model was based on the neural network output feedback method, which does not affect the training process but operates through the prediction model already developed. The logic determining window opening for ventilation used the momentum-based gradient descent method by setting a node with a separate sub-routine that calculates the difference between temperature change after 30 min and the target temperature. This operates through a cost function based on the mean square error (MSE):

(1) $$C_{m}\left(k\right)=\frac{1}{2}e_{m}{\left(k\right)}^{2}$$

where *e_m_*(*k*) is the error between the target temperature and predicted model output. 

**Figure 4:**
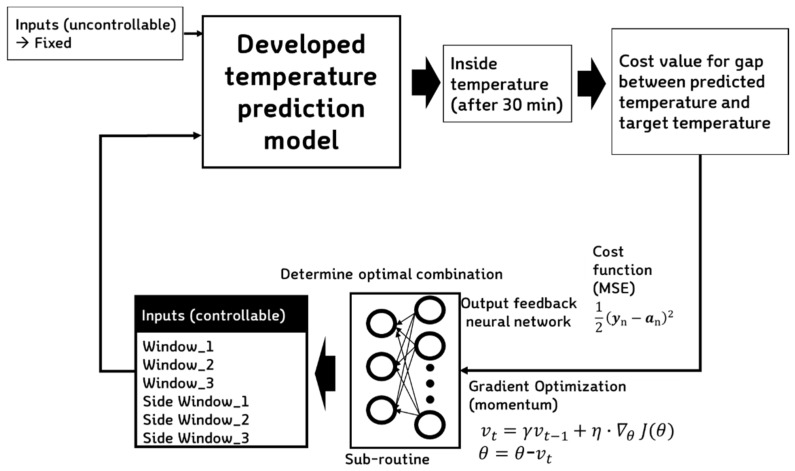
Structure of the output feedback neural network (OFNN) and operational direction.

The proposed logic operates to reduce *e_m_* (*k*) through a separate artificial neural network layer installed to adjust the weight of the neural network nodes (Figure 4). In order to adjust the weight parameter, a momentum term was included, a well-known function used to increase the rate of convergence dramatically [36,37]:

(2) $$\Delta w_{t}=-\varepsilon \nabla_{w}E\left(W\right)+p\Delta w_{t-1}$$

where p is the momentum parameter (0.01 was used). The modification of the weight vector at the current time step depends on both the current gradient and the weight change of the previous step [37].

**Figure 5:**
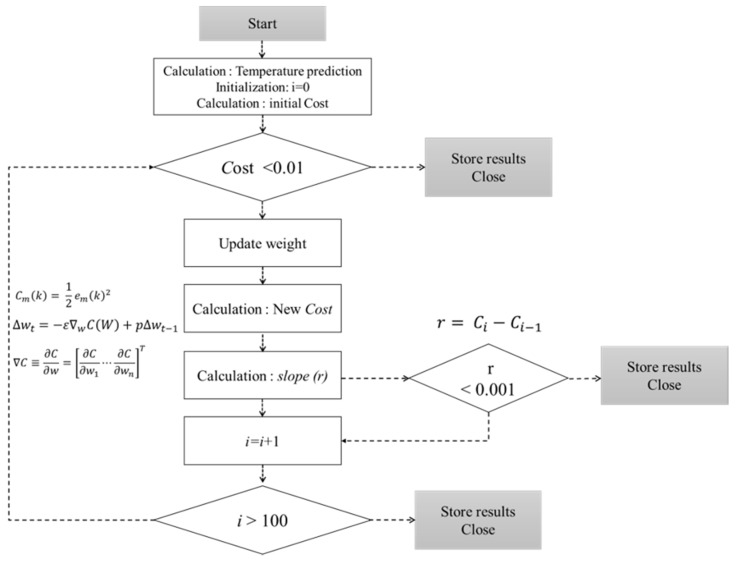
Flowchart for optimal control values using the gradient descent method.

The sub-routine stops repeating if three conditions are met (Figure 5) as follows: (1) when the cost function value calculated by the predicted temperature decreases below a certain value (cost: 0.01), (2) when the progress value (difference between the value before and after the update) decreases below a certain value (r: 0.001), and (3) when the number of iterations reaches the set number of times (i: 100). At this point, the iterations stop, and the process outputs the determined value to the signal corresponding to the opening of six windows. The OFNN operates in conjunction with the predictive model, and the input variables are the MSE calculation results between the target temperature and the current temperature. The hidden node is composed of two layers, each having 15 and 10 nodes, and Tanh is used as the activation function. The output is six nodes, each of which directly determines the window open control signal.

### 2.4. Simulation and Field Experiment Testing

In order to test the new control algorithm, simulations and field verifications were conducted on specific conditions. On May 11, 2018, using the environmental data from three time points on this day, the signal decision process and decision process of the developed algorithm were confirmed. At this time, the predicted temperature and cost value for each loop determined repeatedly were checked, and the decision history about multi-window opening was compared. The final result of this case simulation comparison was to determine the operation of the OFNN-based optimization algorithm in practice. In addition, the result could be confirmed by comparing the relationship between the signal change of the six windows and the effect of the obtained value on the temperature prediction model. The three cases below were selected based on the three most important points of the day, namely after sunrise, after midday, and before sunset. The details of three time points are shown in Table 3. The change of control signal of each window due to OFNN was observed at each time point. In each case, the external and inside environmental conditions were fixed, and the ANN and OFNN models operated repeatedly to determine the six window open signals; the specific environmental conditions are shown in Table 3. In addition, the ANN model and OFNN were simulated simultaneously under the assumption that the whole day was continuously controlled by the proposed method.

**Figure 6:**
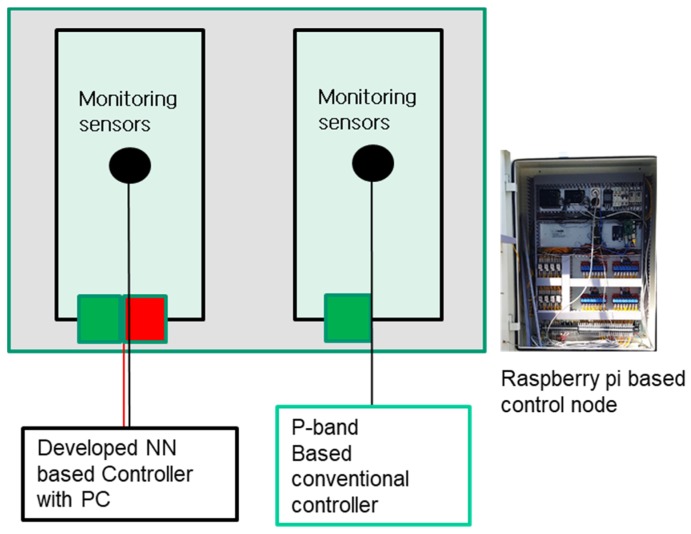
Comparative greenhouse and control node diagram for field application experiment.

Field tests were carried out from May 18 to May 24, 2018, in which two greenhouses were used (Figure 6). The controller equipped with the OFNN algorithm newly developed in this study and a controller equipped with a standard commercial controller were installed in the greenhouse, respectively, and the control performance was evaluated by setting the desired temperature value. The commercial controller adopted the P-band algorithm, which is a kind of proportional parameter that determines the opening angle of the window according to an excess of the desired temperature; the difference between the set point and the measured point is the reciprocal of the proportional gain constant [34,38]. 

For comparison experiments, both controllers used a Raspberry Pi microcontroller, which received environmental sensor information and sent it to the server. In the greenhouse equipped with our control algorithm, an additional computer (Core i7-6700 processor, Intel®, Santa Clara, CA, USA) was installed to infer prediction models and operate subroutines for determining the control signal. 

## 3. Results

### 3.1. Performance of Temperature Prediction Model

**Figure 7:**
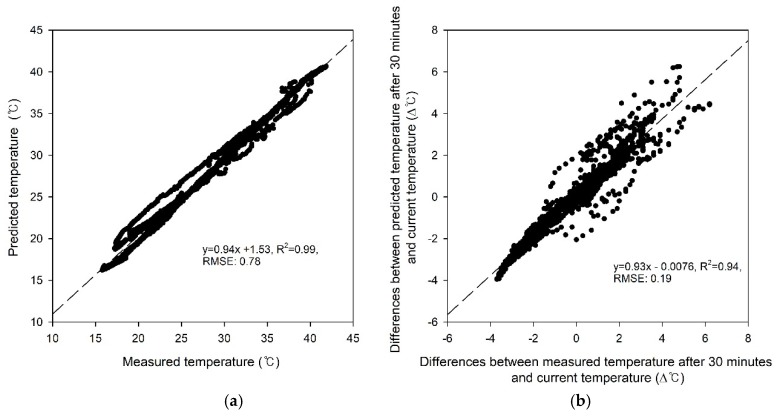
Comparisons between (**a**) predicted and measured temperature and (**b**) difference between the current temperature and predicted or measured temperature after 30 min.

The prediction results for the ~11,000 validation samples in the learning process yielded a 0.99 R^2^ for the calibration curve with a slope of 0.94, an offset of 1.53, and a total RMSE of 0.78 °C (Figure 7a); this showed a very high accuracy of the developed model during training and validation. The temperature changes after 30 min ranged from –3.9 °C to 6.3 °C (Figure 7b); a comparison of the predicted and measured temperature change showed an R^2^ of 0.94 and an RMSE of 0.19. 

### 3.2. Simulation and Field Test Results

**Figure 8:**
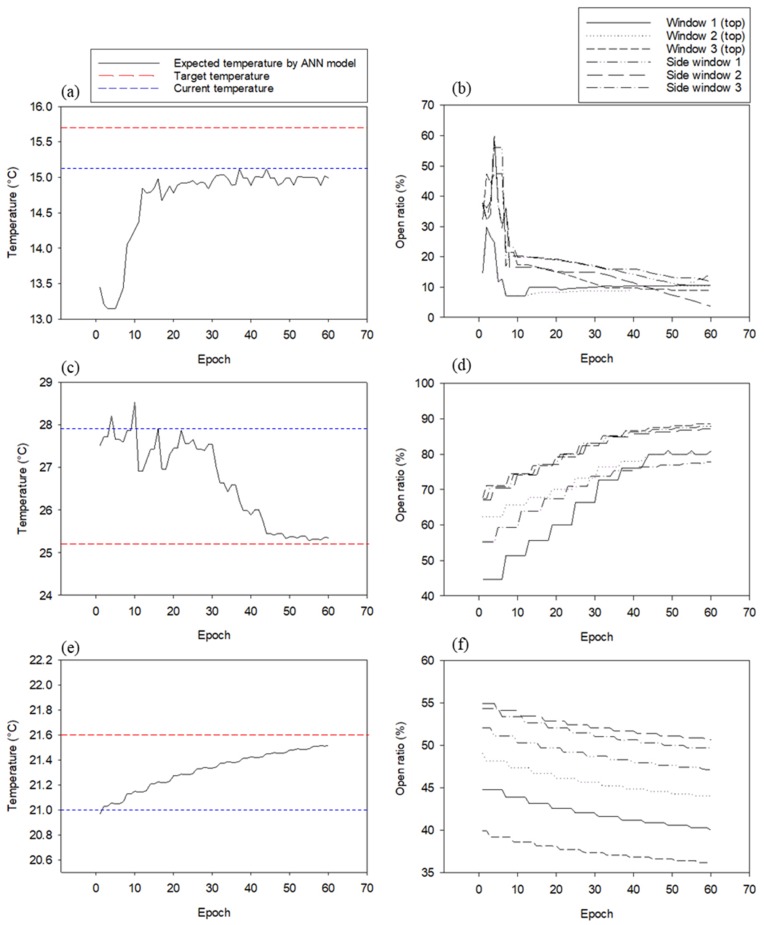
Three cases of predicted temperature change and window open ratio change plot as a result of the operation state of OFNN and ANN during 60 epochs; (**a**) expected temperature changes in case-1; (**b**) changes in window open ratio determined by OFNN in case-1; (**c**) expected temperature changes in case-2; (**d**) changes in window open ratio determined by OFNN in case-2; (**e**) expected temperature changes in case-3; (**f)** changes in window open ratio determined by OFNN in case-3.

**Figure 9:**
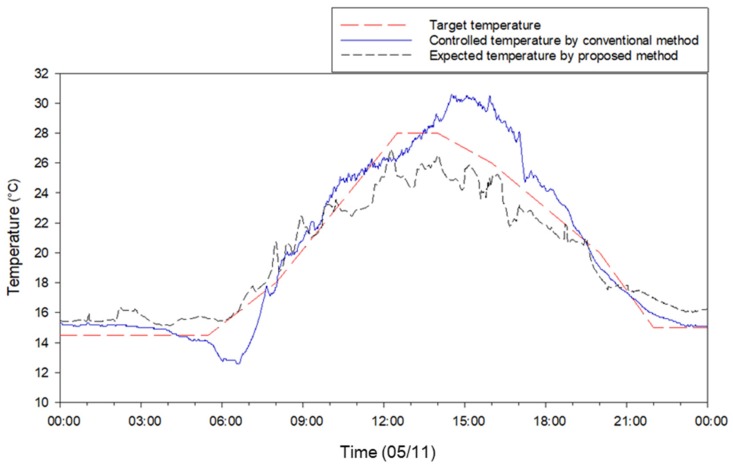
Comparison of temperature control performance between the proposed method and the conventional controller on 11 May.

The OFNN-based control signal decision algorithm proposed in this study first performed driving verification in three cases. Figure 8 shows the expected temperature change (left) and window opening change plot (right) simultaneously with the case-specific OFNN algorithm. Figure 9a,b shows the progression at 6:30, when the sun rises. The red dotted line in Figure 9a is the target temperature, 15.70 °C, and the blue dotted line is the current temperature at that time, which is about 15.08 °C. The initial expected temperature was about 13.40 °C, and the temperature drop was expected below 4 epochs. As seen in Figure 8b, the control algorithm initially inferred that the window was open, but since the expected temperature was far from the target temperature, the process of epoch 5-7 gave the command to close the window. The final expected temperature was 15.04 °C, and six window openings were determined to close by 10%–20%. Figure 8c,d shows the case around 15:30 when the current temperature was about 27.91 °C and the target temperature was about 25.02 °C. As seen in Figure 8d, the six windows converged on the open signal (70%–90%) rather than before. Figure 8 e,f shows that the inside temperature, which rose during the afternoon around 18:30, suddenly dropped as the sun radiation dropped at sunset. The current temperature and the predicted temperature were about 21.0 °C; in order to maintain the target temperature of 21.6 °C, the windows were converging with the closing command.

**Figure 10:**
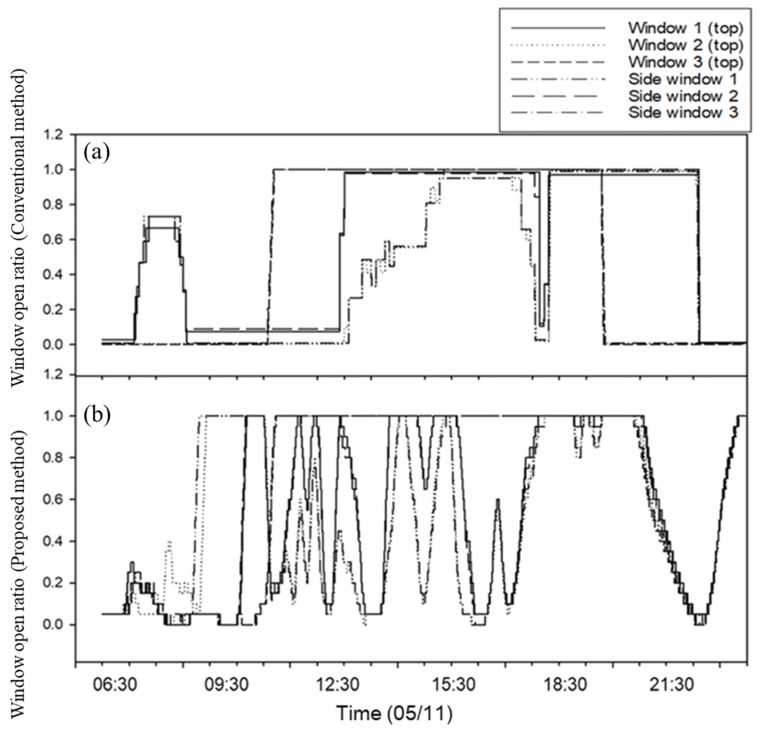
Comparison of window control history for the conventional method (**a**) and the proposed method (**b**) on 11 May.

Based on the case results of ventilation control, the OFNN-based ventilation control for a whole day on 11 May was simulated and compared with the conventional control method in detail (Figure 9 and Figure 10). While the overall RMSE between the target ventilation temperature and the actual measured temperature during the day was ~2.50 °C, this was improved (1.54) with better ventilation control performance when the proposed method was used. A comparison of the opening ratios for six windows under the same environmental conditions on 11 May (Figure 10) showed that the proposed method adjusted windows more frequently or precisely than the conventional controller. Both sets of results demonstrate that the developed algorithm is useful for field applications. 

**Figure 11:**
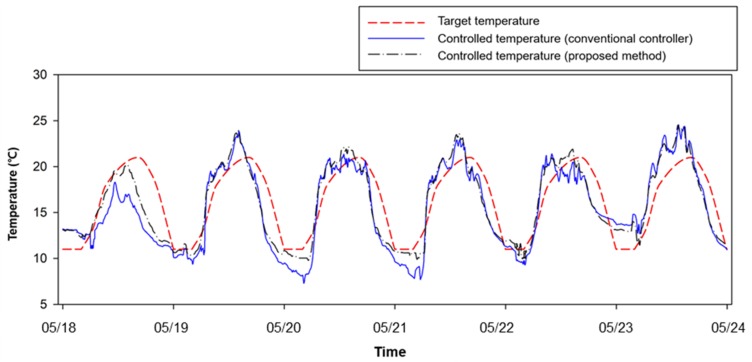
Field test results for the proposed method from 18 May to 24 May.

**Figure 12:**
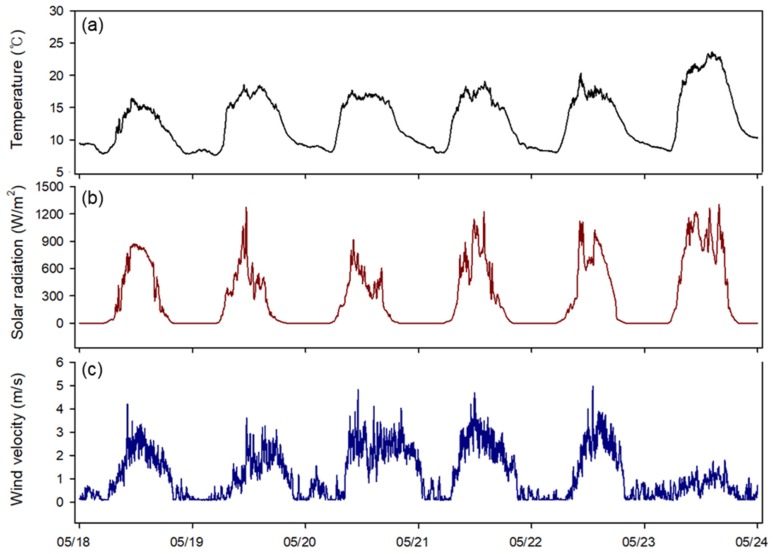
Changes in outside environmental conditions in the experiment: outside temperature (**a**), solar radiation (**b**), and wind velocity (**c**).

**Figure 13:**
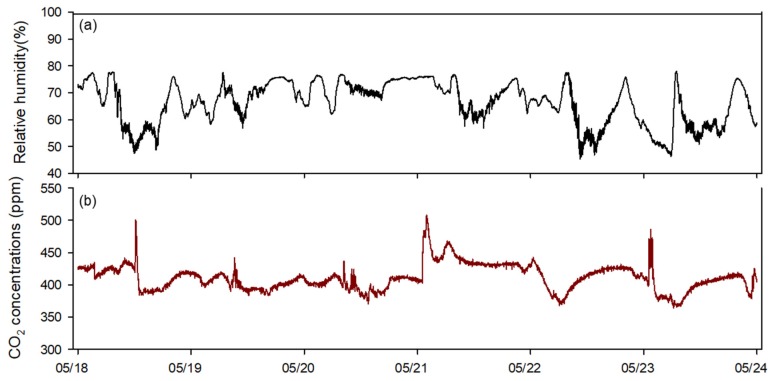
Changes in inside environmental conditions in the experiment greenhouse: relative humidity (**a**), CO_2_ concentration (**b**).

Finally, the OFNN based control logic was mounted on a real strawberry greenhouse, and our proposed logic and conventional ventilation control method were applied to two of the same greenhouses. Over a six-day field test from 18 to 24 May, the RMSE of the target temperature as compared with the conventional controller (3.01 °C) was higher than that for the proposed method (2.45 °C), again confirming the better control performance in the field application (Figure 11). Figure 12 and Figure 13 show changes in the environment outside the greenhouse and other environmental factors inside the greenhouse, respectively. The factors affecting the inside of the greenhouse appeared to have a large proportion of outside temperature and solar radiation. In addition, it was possible to observe changes in the environmental conditions inside the greenhouse and the outside wind velocity during the field test period.

## 4. Discussion

In this study, two main techniques were applied to an effective multi-window ventilation control in the greenhouse. The first, a temperature prediction model based on neural networks, was applied in environmental modeling inside of a greenhouse. Many studies of a predictive model have been positively reviewed on machine learning or deep learning technology in modeling the greenhouse environment [39,40,41]. As shown the results in Figure 7a, the developed model for predicting temperature changes performed well (R^2^: 0.99, RMSE: 0.78), and model-based control was feasible due to the precise predictive models. In addition, it will be applicable to develop prediction models for various time lapses in the future, which will contribute to the improvement of control performance. The second is the control signal decision algorithm through the optimization of the OFNN structure. The non-linear relationship between the temperature change and ventilation rate of six windows was solved by the repeated momentum gradient method. It was determined that this method could be a good guideline for field application in greenhouse environment modeling research. 

P-band-based control logic is mainly used in commercialized greenhouse controllers [34] and requires users to input many setting values for optimal environmental conditions, which is not easy for growers. In contrast, the predictive model and control logic developed in this study were trained by greenhouse conditions without the setting values input separately. For example, in the 11 May test (Figure 9 and Figure 10), the actual temperature decreased sharply between 6 AM and 7 AM, caused by low-temperature air entering the greenhouse. As for the conventional control based on P-band logic, it considered much ventilation due to the influence of increasing solar radiation after sunrise and opened several windows (Figure 10a). This is because a simple linear algorithm makes a decision to open the window in response to a sharp rise in temperature at this time. The control method developed in this study, however, was trained from the greenhouse data; the temperature drop due to window opening was predictable, so the controller decided to keep the window closed at this time and opened a bit (Figure 10b). This effect can be confirmed by the field experiments from 18–24 May. The environmental control method based on the actual applied environmental model was steadily proposed, and it was a major trend of applying the simulation through system identification [42,43]. In this paper, the proposed method was implemented by the proposed optimization method, and the feasibility was confirmed through the field application results. This method is expected to be applicable not only to greenhouses ventilation control but also to ventilation management of livestock facilities or environmental management in residential buildings. This study evaluates that the neural network-based prediction model and control logic yield better control signals only in the greenhouse where training was conducted. In addition, the logic was applied in the external climate of the spring and autumn season, and the window control was performed only for ventilation. However, humidity and radiation are also important factors affecting ventilation. Therefore, various environmental factors and greenhouse structures should be considered by various attempts of OFNN structure and cost functions. 

A server computer was installed in the field due to the overloading of the Raspberry Pi-based micro-controller. The developed logic executed up to 60 sub-routines in determining the control signal while updating the prediction model and the control node. In Raspberry Pi, this took 1100 ± 125 ms while using 100% of the CPU, meaning that environmental monitoring and server data transfer could not be performed at the same time. In addition, the numerical values used in the optimization suggested in the study (cost: 0.01, r: 0.001) could find the optimal convergence conditions in different ranges for other applications, and it is possible to compute the numerical analysis faster by adjusting these values. Solving this problem could be achieved through parallel algorithm optimization or cloud computing technology that implements real-time control algorithms. 

## 5. Conclusions

This study proposed an optimal ventilation control system for managing greenhouse temperature composed of a neural-network-based prediction model and optimization node, then verified our approach through simulations and field experiments.

The prediction model was trained to predict inside temperature 30 min ahead using 15 types of input data parameters. Comparing the predicted and measured temperature yielded an R^2^ of 0.99 and an RSME 0.78 °C in the validation samples; comparing changes in temperature yielded an R^2^ of 0.94 and an RMSE 0.19 °C. These results showed that the developed predictive model successfully forecasted temperature changes in greenhouses and applied it to improved ventilation control. 

An output feedback neural network based optimization method was proposed and implemented with the temperature prediction model. The simulation results confirmed an improved control performance compared to the conventional P-band controller. In addition, a control system based on this logic was used in a field experiment for six days by comparing two greenhouses driven by conventional control logic and the developed control logic; a comparison of the results showed RMSEs of 3.01 °C and 2.45 °C, respectively. This field test clearly demonstrated the superior performance of the output feedback neural network for greenhouse ventilation control. In addition, it was found to be useful in analyzing nonlinearity in black-box based predictive model control. This promising control system can help create optimal control decisions for ventilation and has the potential to be applied to other greenhouse control systems such as irrigation and heating/cooling. In future studies, it is necessary to study the optimization of control parameters and field tests on various greenhouse structures.

## Figures and Tables

**Table 1:** Sensor specifications for the inside climate of the greenhouse.

Component	Measurement Range	Resolution	Operating Temperature (°C)	Response Time (s)
Temperature	–10.0–50.0 °C	±0.3 °C	–25.0–85.0	5.0–30.0
Humidity	0–99.0% RH	±2.0%	–10.0–50.0	8.0
CO_2_	0–3000 ppm	±10.0–50.0 ppm (Proportional to measurement range)	–10.0–50.0	2.0

**Table 2:** Functions used in the artificial neural network hidden nodes.

Name	Equation	Derivative
Tanh	$f\left(x\right)=\frac{2}{1+e^{-2x}}-1$	$f^{\prime }\left(x\right)=\frac{1}{x^{2}+1}$
ReLU	$f\left(x\right)=\{\begin{array}{c} 0\;for\;x<0\\ \;x\;for\;x\;\geq 0 \end{array}$	$f^{\prime }\left(x\right)=\{\begin{array}{c} 0\;for\;x<0\\ \;1\;for\;x\;\geq 0 \end{array}$

**Table 3:** Three case environmental conditions to confirm the simulation of the optimization algorithm.

	External Temperature (E_t_)	External Humidity (E_h_)	Radiation (R_v_)	Wind Speed (W_s_)	Inside Temperature (I_t_)	Inside Humidity (I_h_)	Inside CO_2_ (C_i_)	Time
Case-1	14.1 ℃	64.5%	25 W/m^2^	1.4 m/s	15.2 ℃	61.2%	454.3 ppm	06:30
Case-2	26.9 ℃	62.5%	788 W/m^2^	0.8 m/s	27.9 ℃	67.3%	414.3 ppm	15:30
Case-3	19.5 ℃	71.5%	105 W/m^2^	2.3 m/s	21.0 ℃	55.5%	464.6 ppm	18:30
